# Improved Global Capacity for Influenza Surveillance

**DOI:** 10.3201/eid2206.151521

**Published:** 2016-06

**Authors:** Lauren S. Polansky, Sajata Outin-Blenman, Ann C. Moen

**Affiliations:** Centers for Disease Control and Prevention, Atlanta, Georgia, USA

**Keywords:** influenza, capacity building, surveillance, systems strengthening, global health security, program evaluation

## Abstract

During 2004–2009, the Centers for Disease Control and Prevention (CDC) partnered with 39 national governments to strengthen global influenza surveillance. Using World Health Organization data and program evaluation indicators collected by CDC in 2013, we retrospectively evaluated progress made 4–9 years after the start of influenza surveillance capacity strengthening in the countries. Our results showed substantial increases in laboratory and sentinel surveillance capacities, which are essential for knowing which influenza strains circulate globally, detecting emergence of novel influenza, identifying viruses for vaccine selection, and determining the epidemiology of respiratory illness. Twenty-eight of 35 countries responding to a 2013 questionnaire indicated that they have leveraged routine influenza surveillance platforms to detect other pathogens. This additional surveillance illustrates increased health-system strengthening. Furthermore, 34 countries reported an increased ability to use data in decision making; data-driven decisions are critical for improving local prevention and control of influenza around the world.

## Abstract 2

CDC’s international capacity-building program shows evidence of progress.

After the threat of highly pathogenic avian influenza in 2004, the Centers for Disease Control and Prevention (CDC) began an international capacity-strengthening program with national governments across the globe. The program focused on strengthening 2 systems for preparedness: routine laboratory diagnostics to detect seasonal and novel influenza viruses and routine sentinel surveillance for influenza-like illness (ILI) and severe acute respiratory infection (SARI).

To foster sustainable development, the program prioritized the following principles: investing in routine national surveillance systems to ensure that capacities are regularly tested and used; providing long-term technical assistance driven by country performance and needs; and supporting development that builds on the existing World Health Organization (WHO) Global Influenza Surveillance and Response System. This latter principle includes alignment with WHO guidelines and recommendations for strengthening national laboratory capacities, a requirement for designation as a WHO National Influenza Center (NIC) and for implementation of the 2005 International Health Regulations, a legally binding framework for improving commitment to strengthening core aspects of an infectious disease preparedness and response system (*1**–**3*). Implicit in each principle is respect for the government as the decision-maker, implementer, and beneficiary of the capacity-strengthening process.

The 10-year program is managed through a cooperative agreement between CDC and a country’s ministry of health or equivalent national health agency. The first 5 years of the program’s phased approach focuses on capacity building; over the following 5 years, financial support from CDC is incrementally reduced. Reducing funding encourages transition of financial support for built routine surveillance systems to the countries. Through the cooperative agreement mechanism, the program provides support in 3 ways: providing funding for equipment, materials, and locally employed personnel; conducting hands-on training and long-term technical follow-up with staff within a country; and facilitating participatory, standardized assessments (http://www.cdc.gov/flu/international/tools.htm) of national influenza laboratories, surveillance systems, and core capabilities for influenza pandemic preparedness, each with targeted technical recommendations (*4**–**6*).

Evaluating outcomes of capacity building can be challenging for many reasons, including variation among countries, lag between capacity-building activities and performance outcomes, and methodologic challenges of collecting and analyzing data from multiple countries (*7*). Through systematic review of the funding opportunity announcements, we found the following 6 development areas to be the most emphasized: 1) achieving WHO NIC recognition; 2) improving weekly testing for influenza; 3) maintaining sentinel surveillance in ≥3 sites; 4) reporting weekly data to the WHO FluNet virus monitoring system; 5) sharing specimens with WHO collaborating centers for seasonal vaccine strain selection; and 6) increasing awareness of influenza disease by using national surveillance data to guide decision making for prevention and control strategies. These development areas are the focus of our evaluation.

During 2004–2013, a total of 39 countries participated in the program. We conducted a retrospective evaluation of the extent to which capacity was strengthened in the 6 focus areas after 4–9 years of countries’ participation in the program.

## Methods

We collected data from external WHO sources (*8**–**10*; WHO, unpub. data) and a 2013 retrospective questionnaire that gathered information about capacity indicators from countries that began the program during 2004–2009. Analysis of the questionnaire indicators enabled systematic analysis of information unavailable from WHO sources, including partners’ perspectives of the program. All countries that transitioned from capacity-building to the 5-year sustainability cooperative agreement and returned the completed questionnaire were included in our analysis.

### WHO Data Sources

We analyzed the change in the number of countries designated as WHO NICs; an increase indicates a strengthened global surveillance network. NIC designation depends on several achievements, including the ability to monitor circulating influenza and isolate influenza viruses, a capacity that is key for selecting viruses for vaccines (*11**,**12*). We downloaded public data from the WHO Global Influenza Surveillance and Response System’s FluNet, which monitors circulation of influenza viruses globally. For each country, we calculated the change in total number of specimens processed annually from the time the program started in a country through 2013; results served as a proxy indicator of a country’s ability to collect, transport, and test specimens for influenza (*8*). For the descriptive program data, we calculated median values and interquartile ranges (IQRs). We also calculated the change in the number of countries reporting data on circulating viruses to FluNet for >90% of weeks in each year; this calculation served as an indicator of a country’s ability to collect and share this information routinely on WHO’s global platform (*8*).

In 2007, WHO developed a voluntary External Quality Assessment Project to test the quality of reverse transcription PCR (RT-PCR) diagnostics for influenza (*13*). We analyzed the change in the number of countries that participated in this project and, for participating countries, the number that scored 100% on all panels for each year during 2007–2013; these calculations served as indicators of progress made in the quality of influenza testing (*9*). During 2007–2011, two panels were available each year; during 2012 and 2013, only 1 panel was available.

Each year, the 5 WHO Collaborating Centers for influenza receive influenza specimens or viral isolates from NICs to analyze for seasonal influenza vaccine strain selection (*14**,**15*). Using the Northern and Southern Hemisphere vaccine strain selection information packages, we analyzed the change in the number of countries with NICs that shared specimens at the start of the program, compared with those sharing specimens in 2013; this change served as an indication of global contribution to vaccine strain selection. All data were analyzed by using Microsoft Excel (Redmond, WA, USA).

### Questionnaire

The 2013 retrospective questionnaire was available in English, French, and Spanish in electronic and paper versions and was piloted in 3 countries before implementation. The pilot program included discussions of question interpretation with respondents to ensure consistency in attribute measurement. We analyzed the extent to which countries believed that the program contributed to their ability to collect and report data to WHO FluNet and to prepare for the 2009 influenza A(H1N1) pandemic (pH1N1). Responses used a Likert scale (i.e., critical, major, somewhat, little, none) and described qualitatively the contributions made. We inductively coded the main ideas mentioned and reported them by frequency of mention. To evaluate the growth in surveillance capacity, we analyzed the number of influenza sentinel sites conducting ILI or SARI surveillance and their geographic coverage during the first year of support and compared findings with those data for 2013. We also analyzed questions about additional pathogens that were added to the routine influenza diagnostic testing platforms and other types of syndromic surveillance conducted at influenza sentinel sites. Finally, we analyzed how countries ranked types of CDC program assistance (i.e., direct funding, technical and training assistance, objective assessments of capacity, and information exchange during meetings) on the basis of the programs’ ability to improve functioning of the national surveillance system.

To assess internal validity of the questionnaire, we asked 2 questions for which we had externally validated data as a proxy test. One question asked if the country was reporting to WHO FluNet before starting the cooperative agreement with CDC. The other asked whether the country was sending specimens or viral isolates to WHO Collaborating Centers for influenza seasonal vaccine strain selection before starting the cooperative agreement with CDC. The accuracy of responses to those questions was >90%, indicating that for those 2 questions, history and maturation bias had little effect on the internal validity of responses. Data were double entered and analyzed by using Epi Info version 7.1.2 (CDC, Atlanta, GA, USA).

## Results

Of the 39 countries that partnered with CDC to improve influenza surveillance capabilities, 36 (92%) transitioned to CDC’s 5-year sustainability cooperative agreement, and 35 (97%) completed the questionnaire (Table 1). Among those responding to the questionnaire, 10% had worked in the country’s national influenza programs for 1–3 years; 31% for 4–6 years; 31% for 7–9 years; and 27% for >9 years. No respondent had <1 year of experience.

**Table 1:** Countries that partnered with CDC in capacity strengthening for influenza surveillance, by start year and World Health Organization Region, 2004–2009*

Start year	AFR	EMR	EUR	AMR	SEAR	WPR
2004	–	Pakistan	–	–	India, Indonesia, Thailand	China, Mongolia, Philippines
2006	Angola Democratic Republic of Congo Cote d’Ivoire Ethiopia Nigeria Rwanda South Africa Republic of Tanzania Uganda	Afghanistan Morocco	Armenia Georgia Russia Ukraine	Brazil Mexico	Bangladesh	Vietnam Cambodia Laos People’s Democratic Republic
2009	Madagascar Zambia	Egypt	Republic of Moldova	Paraguay	Nepal Sri Lanka	–
Total	11	4	5	3	6	6

*A total of 39 countries partnered with CDC for influenza surveillance capacity strengthening during different years; 35 countries responded to a 2013 questionnaire and are included in our analysis. AFR, African Region; CDC, Centers for Disease Control and Prevention; EMR, Eastern Mediterranean Region; EUR, European Region; AMR, Region of the Americas; SEAR, South East Asian Region; WPR, Western Pacific Region; –, no countries partnered with CDC during that start year.

### Improved Performance of Influenza Laboratories

**Figure 1:**
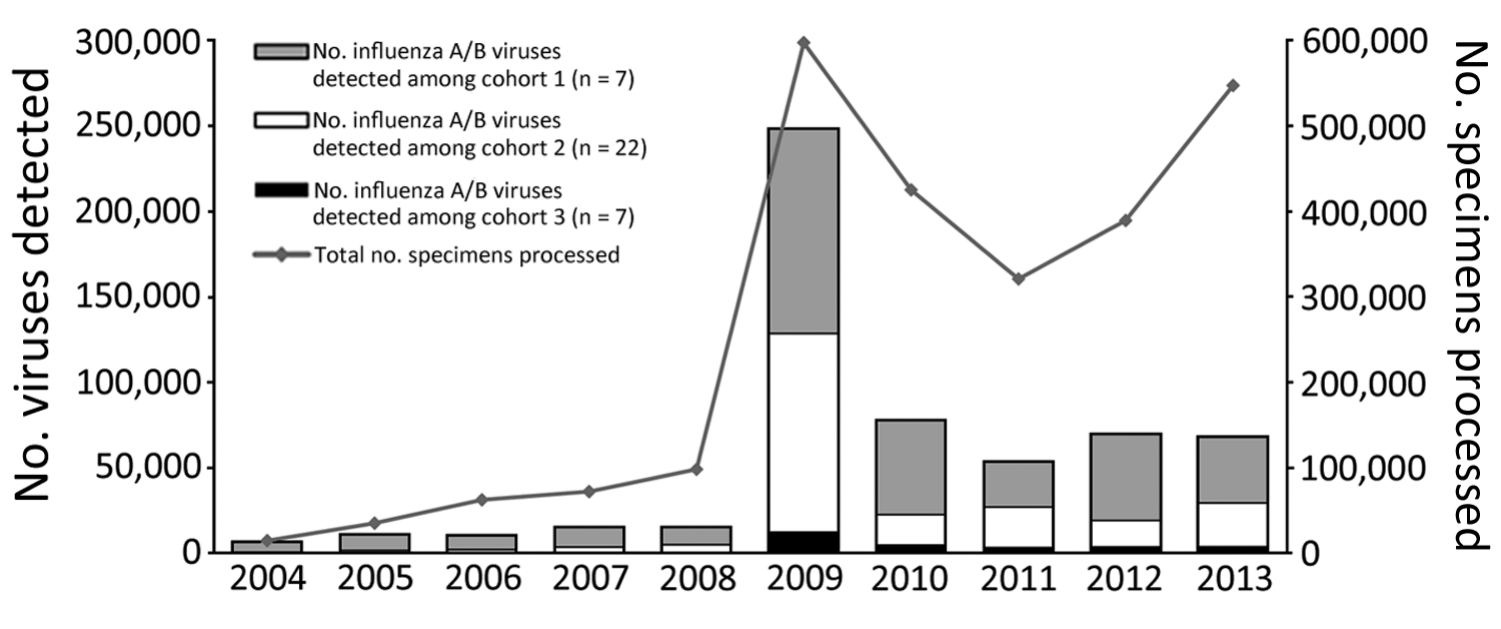
Changes in numbers of influenza specimens processed and in numbers of viruses detected per year among 35 countries that partnered with the Centers for Disease Control and Prevention to strengthen influenza surveillance capacity, 2004–2013. From a total of 39 participating countries, 35 responded to a 2013 questionnaire and are included in this analysis.

**Figure 2:**
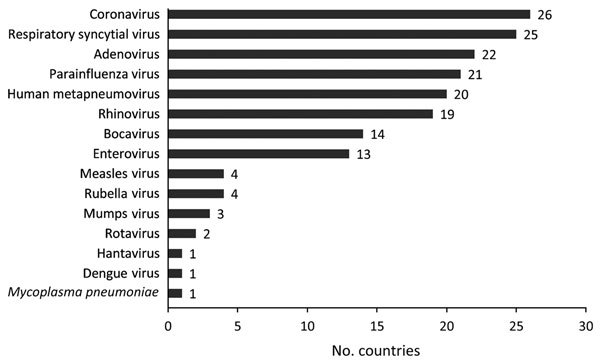
Number of countries that reported adding different virology testing assays to routine influenza laboratory testing platform by virus type from the start of the partnership program with the Centers for Disease Control and Prevention to strengthen influenza surveillance capacity, 2004–2013. From a total of 39 participating countries, 35 responded to a 2013 questionnaire; 28 reported adding tests for other pathogens.

The number of countries conducting routine virologic surveillance for influenza increased from 19 at the start of the capacity-strengthening program to 35 in 2013. The total annual number of specimens tested increased substantially, from 81,851 (median 37, IQR 0–2,411) at the start of the program to 542,235 (median 2,826, IQR 1,282–5,052) in 2013; most growth occurred during the year after the pH1N1 pandemic (Figure 1). Besides having influenza testing, 28 (80%) countries reported adding additional pathogens to the routine platforms that were developed or enhanced through capacity strengthening (Figure 2). Of 19 countries with no NIC at the start of the program, 12 (63%) fulfilled the needed criteria and received official NIC designation. The global influenza surveillance network was also enhanced by the designation of a fifth WHO Collaborating Center for influenza in China in 2009 after this country substantially enhanced the scope of its influenza surveillance system.

All national laboratories supported through the program now use real-time RT-PCR as the primary method to detect circulating influenza. Since WHO developed its EQAP quality assurance test for RT-PCR diagnostics for influenza in 2007, the number of countries using RT-PCR diagnostics increased from 11 in 2007 to 34 in 2013. The percentage of countries with no (0.0) error on the EQAP panels increased from 36% (4/11) in 2007 to 85% (30/34) in 2013.

### Improved Weekly Reporting of Viruses

The proportion of countries reporting data to WHO FluNet for >90% of weeks per year increased considerably during 2004–2013 (Table 2). Among all countries, the median percentage of weeks reported per year increased from a baseline of 21% (IQR 0%–98%) to 100% (IQR 79%–100%) after 5 years of support, when all countries transitioned to the sustainability cooperative agreement. Of the 16 countries not reporting during the first year in the program, 14 provided data every week with 100% completion during 2013.

**Table 2:** Percentage of countries with data available in the WHO FluNet for 90%–100% of weeks per year by country start year in the program, 2004–2013*

Start year	% Countries										
	2004	2005	2006	2007	2008	2009	2010	2011		2012	2013
2004, n = 7	44	33	33	56	67	67	100	100		100	100
2006, n = 21	13	14	22	30	30	30	70	70		77	73
2009, n = 7	29	29	14	29	43	43	71	86	100		100

*A total of 39 countries partnered with CDC for influenza surveillance capacity strengthening during different years; 35 countries responded to a 2013 questionnaire and are included in our analysis. CDC, Centers for Disease Control and Prevention; WHO, World Health Organization.

Thirty (86%) of 35 countries reported by questionnaire that the capacity-strengthening program played a critical (n = 10), major (n = 16), or small (n = 4) role in improving FluNet reporting. At the start of the program, median baseline reporting to FluNet was 0% among those countries reporting that CDC played a critical (IQR 0%–25%) and major (IQR 0%–74%) role. Those reporting that the program played a small (median 76%, IQR 30%–95%) or no (median 83%, IQR 60%– 94%) role had much higher baseline reporting than those countries reporting that the program played an important role in increasing FluNet reporting (Table 3).

**Table 3:** Most commonly reported ways that 35 countries used CDC program support to strengthen their ability to report national virologic data to WHO FluNet on a weekly basis, by rank order*

Methods used
1. Establishing reverse transcription PCR capabilities
2. Enhancing electronic database management (i.e., computer, internet, database, software, Web site)	
3. Training database managers, laboratory managers, and laboratory diagnostic technicians	
4. Developing a standardized weekly national report with indicators	
5. Establishing a laboratory focal person to liaise with the sentinel network

*A total of 39 countries partnered with CDC for influenza surveillance capacity strengthening during different years; 35 countries responded to a 2013 questionnaire and are included in this analysis. CDC, Centers for Disease Control and Prevention; WHO, World Health Organization.

### Improved Participation in WHO Influenza Vaccine Strain Selection

The number of countries that contributed isolates or specimens for inclusion in global vaccine strain selection increased from 16 (42%) at the start of the CDC program to 28 (80%) in 2013. Questionnaire responses also showed progress in use of WHO selection criteria; a comparison of data for the start year and 2013 showed that an increased number of countries that selected viruses by age group (7 vs. 18), geographic area (9 vs. 26), phase of influenza season (10 vs. 22), or other high priority criteria for the country (7 vs. 14).

### Growth of Influenza Sentinel Surveillance

On the basis of 35 countries’ responses to the 2013 retrospective questionnaire, 32 (94%) countries established >3 surveillance sites since their start year. The number of sites capable of collecting weekly specimens and epidemiologic data from patients seeking healthcare for ILI or SARI increased from 446 at the start of the program to 2,075 in 2013 (Table 4). Moreover, 48% of countries that began the program with no influenza sentinel sites had 1,293 (median 7, IQR 5–14) functional sites in 2013. The number of provinces or districts with a functional influenza sentinel site increased in 29 (83%) countries.

**Table 4:** Countries with sentinel sites capable of collecting specimens ≥1 time per week from patients screened for ILI or SARI in WHO regions*

WHO region	Start year			2013		
	No. countries with ≥1 site	Total no. sites		No. countries with ≥1 site	Total no. sites	Total increase in sites
AFR	3	174		11	231	57
EMR	3	126		4	176	50
EUR	0	0		5	94	94
AMR	2	60		3	807	747
SEAR	6	55		6	83	28
WPR	3	31		6	684	653
Total	17	446		35	2,075	1,629

*A total of 39 countries partnered with CDC for influenza surveillance capacity strengthening during different years; 35 countries responded to a 2013 questionnaire and are included in our analysis. AFR, African Region; AMR, Region of the Americas; CDC, Centers for Disease Control and Prevention; EMR, Eastern Mediterranean Region; EUR, European Region; ILI, influenza-like illness; SARI, severe acute respiratory infection; SEAR, South-East Asian Region; WHO, World Health Organization; WPR, Western Pacific Region.

**Figure 3:**
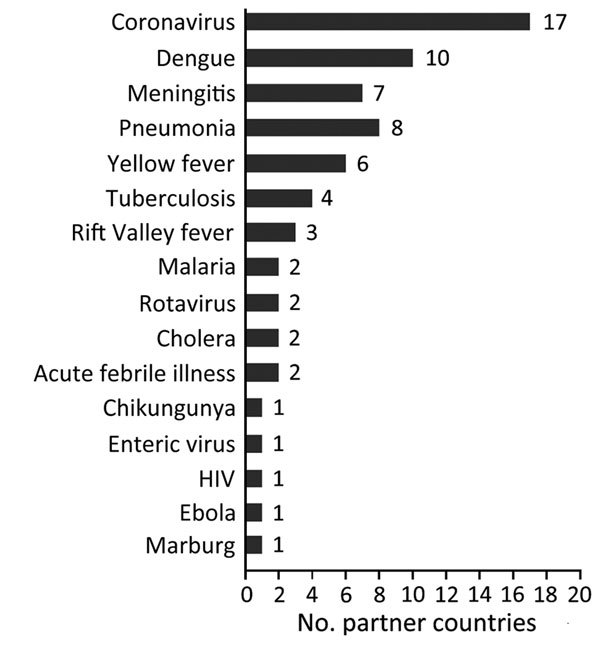
Number of countries that used influenza sentinel sites to initiate surveillance for other infectious diseases or syndromes since the start of the partnership program with the Centers for Disease Control and Prevention to strengthen influenza surveillance, 2004–2013. From a total of 39 participating countries, 35 responded to a 2013 questionnaire; 29 reported initiating surveillance for other diseases or syndromes.

Questionnaire responses for 29 (83%) countries indicated that influenza sentinel sites initiated surveillance for other diseases or syndromes (Figure 3). All 29 reported that program funds and technical assistance played a critical (n = 4), major (n = 16), or small (n = 9) role in capacity building for additional surveillance.

### Improved Country Response to Influenza

Among the 35 responding countries, 26 started the capacity-strengthening program before onset of the pH1N1 outbreak; 25 (97%) of the 26 believed that capacity strengthening played a critical (n = 16) or major (n = 9) role in their pandemic response. In an inductive analysis of capacities that countries reportedly experienced as key to outbreak detection and pandemic response, the most common was establishment of routine sentinel SARI or ILI surveillance systems (Table 5). Also, 15 countries noted improvements to influenza laboratory diagnostics, which made possible identification of pH1N1, highly pathogenic influenza A(H5N1), and influenza A(H7N9) viruses. Other key capacities described were the ability to understand seasonal trends, establishment of subnational diagnostic laboratories, and creation of systems for information sharing between laboratories and sentinel surveillance sites.

**Table 5:** Country perspectives on the role of surveillance system capacities during the 2009 influenza A(H1N1) pandemic in countries with 0–1 influenza sentinel sites at the start of the program*

WHO region	Perspective
South-East Asia	“The first fatal case during the pandemic was identified by our [influenza] network as part of hospital based surveillance activities. The network took a frontline role in providing diagnostics to the respective regions, actively participated in pandemic mitigation activities in coordination with the regional health authorities.”
African	“The cooperative agreement has created awareness of influenza virus among health workers, policy makers and communities at large [and] laboratory capacity to test the virus: before there was no idea if the virus [was] in existence in the country, the types and subtypes, or the staff capacity to identify and respond to influenza.” “The use of the case definitions for influenza-like illness (ILI) and severe acute respiratory infection (SARI) and the virologic analysis of the samples from cases has helped in identifying the onset of the pandemic flu H1N1 in the population and the period of dominance which informed the type of control measures put in place.”
Eastern Mediterranean	“We routinely collect data on ILI and SARI cases. The sentinel sites send the epidemiologic data and specimen to [our] NIC [National Influenza Centre] for verification. We have certain examples of SARI outbreaks that the system easily detected and responded to.”
European	“Sentinel sites are [now] located [along] bird migration routes and near the countries [‘] points of entry. Established SARI case-based surveillance with lab confirmation is very helpful in order to provide timely detection and response to abnormal influenza.”
Western Pacific	“Routine surveillance of epidemiology and viruses provided data on circulating strains with epi-clinical information which helped to detect abnormal influenza and thus helped to implement a plan.”

*A total of 39 countries partnered with CDC for influenza surveillance capacity strengthening during different years; 35 countries responded to a 2013 questionnaire and are included in our analysis. South East Asia Region had 1 sentinel site at the beginning of the program; all other regions had no sites at program start. CDC, Centers for Disease Control and Prevention; WHO, World Health Organization.

### Improved Knowledge of Influenza for Local Decision Making

Among 35 responding countries, 29 reported that they have described the seasonality of influenza viruses in their country; 19 (66%) were described for the first time during the program (Table 6). Thirty-four (97%) countries reported an improved ability to use national influenza data in decision making in several ways: drive updates to national pandemic preparedness plans, create evidence-based vaccine guidelines, determine best use of antiviral medication, and determine need for community mitigation measures such as school closures. Countries in each of 6 WHO regions reported that they used their national surveillance data to support influenza vaccination programs.

**Table 6:** Progress in national estimates and recommendations tied to availability of routine national influenza surveillance data for 35 countries participating in CDC influenza surveillance capacity strengthening, 2004–2013*

Measures	Completed†		In progress‡	Not yet available
	Before	During		
Seasonality	10	19	4	2
Burden of influenza disease among sentinel sites	1	8	17	9
Risk factors for influenza disease	3	4	14	14
Burden of influenza disease in population	1	4	10	20
Antiviral recommendations	7	17	0	10
Vaccine recommendations	9	8	3	15
Vaccine acceptability	3	6	3	23

*A total of 39 countries partnered with CDC for influenza surveillance capacity strengthening during different years; 35 countries responded to a 2013 questionnaire and are included in our analysis. CDC, Centers for Disease Control and Prevention. †Completed means that the measure was completed before or during the 10-year CDC capability-strengthening program. ‡In progress means that the measure was in progress during the 10-year program.

### Development and Ownership of Capacity Strengthening

Overall, 34 (97%) countries reported that they were mostly or very able to meet their countries’ needs through the program; 32 (91%) mostly or always perceived that ownership of the capacity building was theirs. Countries had different perceptions of the program’s impact on development of laboratory versus sentinel site systems. For 29 (81%) of countries, the top-ranked type of assistance for strengthening laboratories was financial assistance for laboratory equipment, materials, and reagents. For the remaining 6 (19%) countries, the most critical assistance was staff training and technical advice (n = 4) and the ability to exchange experience with colleagues during national or international meetings (n = 2). Objective assessments of the laboratory were ranked, on average, as the third most critical assistance.

Rankings regarding strengthening sentinel surveillance differed among countries. Financial assistance was ranked by 17 (49%) countries as most critical. The most critical assistance among the remaining 18 (51%) countries was trainings for staff and technical advice (n = 11), objective assessments of the surveillance system (n = 4), and the ability to exchange experience with colleagues during national and international meetings (n = 3). In the analysis of recommendations suggested in the questionnaire, the most common was to increase technical assistance for assessing, evaluating, and improving the sustainability of capabilities developed.

## Discussion

In the context of the emergence and reemergence of severe acute respiratory syndrome and highly pathogenic influenza A(H5N1) virus, CDC’s Influenza Division developed an international capacity-strengthening program that enabled countries to detect seasonal and pandemic influenza viruses and to make evidence-based decisions for risk reduction (*16**–**18*). Among 35 participating countries included in our evaluation, all indicators examined by using WHO data sources have shown dramatic improvement.

The increase in influenza testing since the start of the program may be driven in part by the growth in ILI and SARI sentinel sites that collect weekly samples. Increases in influenza testing and number of surveillance sites call into question the notion of efficiency: how much surveillance and laboratory testing is enough, particularly in low-income countries where resources are scarce? In the United States, the Influenza Virologic Surveillance Right Size Project was launched in 2010 to help determine the optimal amount of surveillance needed to meet virologic surveillance objectives. This project and other such tools are critical for countries with limited resources and are important for the sustainability of influenza surveillance programs (*19*). As a step towards determining the optimal amount of needed surveillance, the capacity-strengthening program is helping partners clarify their national objectives and evaluate their influenza surveillance systems’ data quality, flexibility, simplicity, stability, acceptability, and utility through training and technical assistance (*20*).

The program’s role in supporting FluNet reporting was perceived as greater in countries that submitted reports during fewer weeks at the start of the than those that reported weekly or almost weekly. This emphasis on the program’s role in increased reporting suggests a greater impact of capacity strengthening in countries with a lower baseline ability to report circulating viruses to FluNet.

Of 35 participating countries, 32 (91%) partly attributed their ability to respond to the pH1N1 pandemic to prior capacity strengthening; this perception of the role of capacity strengthening confirms the critical need for routine clinical, epidemiologic, and virologic influenza surveillance as a preparedness and response strategy. The value of routine surveillance capacity in supporting demands placed on systems during pandemics aligns with previous reports that showed significant progress in core capabilities for influenza pandemic preparedness among the same countries (*5**,**6*). In Bangladesh, enhanced surveillance of laboratory-confirmed pH1N1 infection facilitated a response weeks before the spread to the general population (*21*). In the African region, several countries were able to show the first introduction of pH1N1 virus within their countries (*22**,**23*).

Influenza viruses are constantly changing, requiring updates to the vaccine each year on the basis of which influenza viruses are infecting persons around the world, how those viruses are spreading, and how well the previous season’s vaccine protects against those viruses. Therefore, the increase in the number of countries submitting specimens for seasonal vaccine strain selection is critical for selecting the most representative strains currently circulating.

The recent emergence of Middle East respiratory syndrome coronavirus in Saudi Arabia (*24*) and the devastating outbreak of Ebola in West Africa (*25*) have tested the flexibility of existing surveillance platforms in responding to emerging public health threats. Our findings provide preliminary evidence that existing health systems’ strengthened influenza surveillance capacity, aimed at detecting clinical illness and prioritized for laboratory testing, has facilitated surveillance for other diseases, including Middle East respiratory syndrome and Ebola. More research regarding how influenza surveillance platforms are best leveraged is needed for the future.

Scientific data, such as laboratory-confirmed disease surveillance, aid countries in making evidence-based decisions about influenza preparedness, prevention, and control (*26*). This outcome was reported in 34 (97%) of 35 countries and shows the value of capacity strengthening. In India, surveillance data identified regional differences in the onset and length of influenza seasons; these differences affect vaccine formulation and timing (*27*). Similar evidence has been used in Southeast Asia countries, where progress in surveillance and viral typing has shown year-round circulation in some countries and biannual peaks of circulation in others; these findings informed vaccination recommendations and determination of appropriate timing for vaccination (*28*). Experience from the WHO Region of the Americas shows that the capacity for collecting and using accurate national data leads to more sustainable vaccine programs (*18*).

The results of our evaluation have helped define future focus areas for the program. With the enhancement of influenza surveillance and situational awareness, CDC has developed a new program to support countries wanting to develop vaccination programs around such evidence. The challenge in answering questions about the burden of influenza disease and risk factors (Table 6) illustrates that another key next step will be to ensure that high quality surveillance data and capacity exist to help answer these questions.

That 32 (91%) countries felt ownership of capacity-strengthening offers encouraging evidence for the program’s approach. The perception of increased effects of funding on laboratory strengthening, compared with increased effects on sentinel surveillance, is unsurprising, given the costs of maintaining a laboratory, a well-known barrier to routine surveillance. What is arguably more surprising is the perceived value of technical assistance beyond funding. Some responding countries perceived training and technical advice from experts, objective assessments of capacity, and the ability to share experience as having even greater effects than funding. This finding highlights the need for technical guidance, training, and partnership-building, all of which go beyond basic funding. A review of 8 Central America countries that reported a significant positive correlation between cumulative funding and technical assistance with pandemic preparedness progress supports this finding (*6*).

Strengthened influenza surveillance and detection help countries comply with WHO International Health Regulations and contribute to core competencies under the Global Health Security Agenda, which seeks to improve infectious disease detection, assessment, and response, particularly for novel influenza with pandemic potential (*29*).

The biggest limitation to this study was the reliance on retrospective data. Although WHO’s externally validated data served to increase the validity of the findings, those data also have limitations. Because of the retrospective nature of the analysis, assessing lags in data availability on WHO FluNet each week was not possible, although this assessment assists in understanding the timeliness of monitoring. By their nature, retrospective questionnaires can be problematic because they rely on institutional memory and experience; however, respondents had a relatively high tenure in their national influenza programs. Most (31/35 [89%]) had >4 years of experience, and 20 (58%) had >7 years. The effects of influenza seasonal variation on increases in demand for testing need further elucidation and may be helped by projects such as determining optimal amounts of surveillance needed. Capacity-strengthening gains cannot be precisely attributed to the cooperative agreement because capacity strengthening is complex and involves many systems, organizations, and behaviors beyond the scope of this article.

In conclusion, considerable progress has been made in laboratory and sentinel surveillance capacities, which have proven to be essential building blocks for knowing which strains of influenza circulate globally, detecting and preparing for novel and pandemic influenza, understanding respiratory illness associated with influenza, and expanding public health surveillance beyond influenza. Countries are translating these capabilities into better decision making for their influenza prevention and control programs. Their ownership of capacity building makes this approach an important model for efforts to enhance global detection and response to emerging infectious diseases such as influenza.
