# Correction: Vol. 22, No. 6

**DOI:** 10.3201/eid2210.C32210

**Published:** 2016-10

**Keywords:** errata, erratum, correction

The name of Wenqing Zhang was misspelled in the acknowledgments of Improved Global Capacity for Influenza Surve (L.S. Polansky et al.). The article has been corrected online (http://wwwnc.cdc.gov/eid/article/22/6/15-1521_article).

